# Are portable ankle brachial pressure index measurement devices suitable for hypertension screening?

**DOI:** 10.1371/journal.pone.0283281

**Published:** 2023-03-21

**Authors:** Justyna Janus, Jennifer K. Nicholls, Edward Pallett, Matthew Bown, Emma M. L. Chung

**Affiliations:** 1 Department of Cardiovascular Sciences, University of Leicester, Leicester, United Kingdom; 2 Department of Medical Physics, University Hospitals of Leicester NHS Trust, Leicester, United Kingdom; 3 National Institute for Health Research Leicester Biomedical Research Centre, Leicester, United Kingdom; 4 School of Life Course Sciences, King’s College London, London, United Kingdom; Majmaah University College of Applied Medical Sciences, SAUDI ARABIA

## Abstract

**Objective:**

In a large-scale population cardiovascular screening programme, peripheral artery disease (PAD) and hypertension would ideally be rapidly assessed using a single device. The ankle-brachial pressure index (ABPI) is calculated by comparing the ankle and brachial blood pressure (BP). However, it is currently unclear whether brachial BP measurements provided by automated PAD screening systems are sufficiently accurate for simultaneous hypertension screening.

**Methods:**

Two portable PAD screening devices, the MESI ABPI MD and Huntleigh’s Dopplex ABIlity, were evaluated following the European Society of Hypertension International Protocol (ESH-IP) Revision 2010 using a mercury-free sphygmomanometer as a reference device.

**Results:**

On average, the MESI slightly underestimated brachial systolic blood pressure (BP) with a bias and standard deviation (SD) of -3.5 (SD: 3.3) mmHg and diastolic BP with a bias of -1.5 (SD: 2.3) mmHg. For systolic BP estimates, the Dopplex was more accurate than the MESI with a lower bias of -0.5 (SD: 4.2) mmHg but less precise. The MESI successfully fulfilled all the requirements of the ESH-IP for hypertension screening. The Dopplex device failed the ESH-IP due to the absence of DBP measurements.

**Conclusions:**

The MESI device appears to be suitable for simultaneous PAD and hypertension screening as part of a preventative care programme. Huntleigh’s Dopplex ABIlity failed to pass the ESH-IP validation test. Further clinical trials are underway to assess the use of the MESI for simultaneous screening for hypertension and PAD in a population screening setting.

## Introduction

Peripheral arterial disease (PAD) is a chronic disease resulting from the narrowing of the arteries in the legs due to atherosclerosis [1]. PAD is underdiagnosed worldwide, with at least 50% of PAD patients being asymptomatic [2, 3]. PAD is often associated with hypertension, so early diagnosis of PAD, and better management of high blood pressure (HBP) can improve preventive cardiovascular care, reducing the burden on healthcare providers.

To detect PAD, systolic BP is measured at the posterior tibial artery of the ankle and compared to the systolic BP measured at the brachial artery in the upper arm; the ratio of these measurements is known as the ankle-brachial pressure index (ABPI). If the ABPI is abnormal (<0.9), then the presence of PAD is indicated [4, 5]. Traditionally, ABPI is measured using a handheld continuous-wave Doppler instrument [6] to guide the user in accurately determining the systolic BP. However, this method is time consuming and requires a skilled operator [7].

The recent introduction of automated PAD screening devices means that ABPI measurements can be completed faster than measurements using traditional hand-held Doppler, after minimal user training [8]. This may make it economically feasible to add PAD screening to existing national screening programmes, such as the abdominal aortic aneurysm (AAA) screening programme, which is one of 11 population screening programmes offered in the United Kingdom (UK).

As automated PAD devices use Blood Pressure measurements to estimate the ABPI, automated PAD screening devices could potentially be used to simultaneously check for high BP. However, as separate validated Blood Pressure monitoring devices are readily available, portable PAD screening devices developed for ABPI measurements are generally not licensed or approved for identifying high BP. Consequently, it is unclear whether PAD devices are suitable for identifying high BP, and the accuracy of PAD screening devices for hypertension screening has yet to be assessed.

In this study, two automated PAD devices are assessed for suitability and accuracy for detecting hypertension. The results of this study will inform the design of a combined AAA-PAD-HBP population screening test to be offered to all men in the UK at the age of 65. When designing and evaluating the cost effectiveness of large-scale population screening programmes, it is essential to keep the screening test as short as possible and to minimise equipment costs. If a single PAD device is suitable for combined PAD and hypertension screening, this would be more time and cost effective than conducting separate blood pressure and PAD measurements using different devices.

## Methods

### PAD devices

Two brand new (box fresh) automated PAD detection devices, with valid manufacturer calibration certificates, were tested in this study (S1 Fig and S1 Table). Systems were tested within 3 months of delivery and were purchased in June 2021. Throughout this manuscript, these are referred to as `the MESI’ (MESI ABPI MD system, MESI Ltd., Slovenia, EU), and `the Dopplex’ (Huntleigh Dopplex ABIlity Automatic ABPI system, Huntleigh Healthcare Ltd., Cardiff, Wales).

These devices were purchased as part of a larger UK screening trial [9]; “Peripheral arterial disease, High blood pressure and Aneurysm Screening Trial” (PHAST) designed to test the feasibility of adding combined PAD and hypertension screening to the UK’s existing abdominal aortic aneurysm screening programme.

Both devices were validated for hypertension screening following the European Society of Hypertension International Protocol (ESH-IP, Revision 2010) [10]. Neither of these two PAD devices had previously been evaluated for hypertension screening.

### Nissei DM-3000

A mercury-free sphygmomanometer, Nissei DM-3000 (Nissei DM-3000, Nissei Japan Precision Instruments, Gunma, Japan), acted as a reference device due to the phasing out of the usage of mercury devices within the UK National Health Service (NHS) due to environmental concerns [11]. The Nissei DM-3000 is a validated BP measurement system [10]. It has been previously found to comfortably pass all ESH-IP validation requirements with a similar level of accuracy as a mercury sphygmomanometer, confirming its use as a reliable alternative reference device [12].

The Nissei has two modes; in the automated oscillometric mode, inflation and deflation of the cuffs is fully automated [12]. The automated oscillometric BP mode was selected with the deflation rate set to 2.5 mmHg per second to reduce any variability associated with manual measurements.

The Nissei device has a liquid crystal display, which displays systolic BP, diastolic BP and pulse rate. Two cuff sizes were available for use: standard and large. The University Hospitals of Leicester Clinical Engineering Scientific Services team regularly checked the device’s accuracy using a calibrated pressure meter [13].

The manufacturer’s instructions for the PAD and reference devices were strictly followed to eliminate factors that could impact measurement accuracy [13–15].

### Participants and recruitment

Participants were recruited according to a protocol approved by the University of Leicester Medicine and Biological Sciences Research Ethics Committee and following the Declaration of Helsinki (2013) [16]. All participants provided written informed consent. Each validation study required 33 participants aged over 25, comprising at least 10 men and 10 women. Participants with a history (or family history) of venous thromboembolic disease, limb ulceration, Parkinson’s disease, severe PAD, lymphedema, or clinical evidence of cellulitis were excluded. Participants were excluded if they could not remain still or lie flat, or reported any condition preventing both arms from being measured. Participants taking medication for pulmonary hypertension were included in this study.

Healthy volunteers were asked to refrain from consuming caffeine or nicotine for at least 1 hour before their appointment to avoid transient BP changes. Participants were also asked to avoid vigorous exercise an hour before the study and to wear loose clothing to allow access to their lower limbs and upper arms. The age and sex of the participants were recorded. The correct cuff size was selected by measuring the circumference of the arm. Socks and shoes were removed before measurements. Cuffs were then applied according to the device manufacturer’s instructions. If no suitable cuff size was available, participants were excluded from the study.

### Study protocol

Each participant lay supine on a couch with their back straight and legs uncrossed, resting for 10–15 minutes before the first measurement. Care was taken to ensure that the participant’s heels rested fully on the couch, as placing weight on the calf may affect measurement results. Subjects were asked to remain still and avoid talking. Phones and other devices were removed to avoid interruptions [10, 17].

The validation team included two individuals (an observer and a supervisor) trained in taking BP measurements. The same individuals acted as observer and supervisor for all observations. The accuracy of each PAD device (MESI or Dopplex) was estimated based on comparing BP readings with those from a reference sphygmomanometer (Nissei). Two entry BP measurements were obtained to determine the participant’s suitability for inclusion in the study. One measurement using the reference device (left arm) and one measurement using one of the two automated PAD devices being trialled. For the Nissei measurements, the lower end of the cuff was placed 2 cm above the antecubital fossa and tightness was assessed by placing a finger between the arm and the cuff; two fingers should be able to fit but would be snug. The cuff was inflated to 180 mmHg, and the BP was recorded.

For the MESI device, three cuffs were placed on the participant. One cuff was placed on the left upper arm 2 cm above the antecubital fossa, so the cuff was lined up against the brachial artery, and the other two cuffs were placed 2 cm above the ankle, lined up against the dorsalis pedis artery.

For the Dopplex device, each upper arm chamber was secured in the same way as for the standard sphygmomanometer, with the additional lower chamber attached below the elbow, on the forearm. Using the lower chamber, leg cuffs were secured above the ankle and around the foot. Arrows on all cuffs were pointed upwards to determine the correct orientation of each cuff.

Depending on the entry measurement results from the sphygmomanometer and the device, and the required BP range, participants were included or excluded in the study. If participants were included, the left arm cuff was switched between the sphygmomanometer and the trialled device. Seven BP measurements were taken, alternating between the reference device (four times) and MESI or Dopplex device (three times).

The interval between BP measurements was at least 30–60 seconds to avoid congestion, but no longer than 60 seconds apart as natural variations in BP are likely to occur over extended periods [10]. The total time for obtaining all seven measurements was approximately 60 minutes.

Mean values obtained from the reference device were used to classify each subject’s systolic and diastolic BP as low, medium, or high (S2 Table). Subjects were excluded if the test and reference device failed to record a measurement after three successive attempts. If high BP was identified and the subject was unaware, a consultation with their general practitioner was advised.

### Data analysis

BP readings were analysed as outlined by the ESH-IP validation protocol [10]. This involved comparing the test device BP measurement with measurements made before and after the test device using the reference device. The smallest difference between the reference and the test device was taken forward for further analysis. This resulted in three pairs of reference and test device readings relating to the systolic BP. In the case of the MESI, the diastolic BP of each participant also resulted in three pairs of readings. Each of these 6 readings was classified into 4 groups; within 0–5 mmHg, 6–10 mmHg, 11–15 mmHg, or >15 mmHg of the reference reading [10].

### Statistical analysis

Statistical analysis was performed with GraphPad Prism 9.1.2 Software (GraphPad Software, Inc., San Diego, CA). Continuous parameters were checked for normality and are reported as a mean and Standard Deviation (SD). Bland-Altman analysis of the systolic and diastolic BP readings were used to estimate the bias and 95% limits of agreement of the test device relative to the reference device. The relationship between the test and reference measurements was summarised by fitting a straight line using simple linear regression. Pearson’s coefficient of correlation (R^2^) was calculated and considered to indicate a high correlation for values >0.9.

## Results

Thirty-five participants were screened using the MESI, and thirty-four were screened using the Dopplex device. Two participants screened using the MESI device and one using the Dopplex device were excluded. For the MESI device, one of the participants was excluded due to persistent inflation errors. The other participant was excluded due to the absence of displayed values after inflation. For the Dopplex device, one participant was excluded due to absent values for all 4 limbs. This resulted in a total of 33 participants with readings suitable for further analysis (S3 Table). Although studies for the 2 devices were conducted separately, 30 participants volunteered for both studies, and demographic characteristics were almost identical for evaluation of both test devices (Table 1).

**Table 1:** Participant demographic data for both devices.

	MESI ABPI	Dopplex ABIlity
**Male: Female**	18: 15	18: 15
**Mean Age (SD, range)**	55 (19, 25: 87)^a^	54 (20, 25: 87)^a^
**Mean Arm circumference (cm) (SD, range)**	30.0 (3.4, 24: 38)	30.0 (3.4, 24: 38)
**Cuff for the test device (Standard)**	29	29
**Cuff for the test device (Large)**	4	4

^a^ The slight difference in the participants’ mean age is due to different individuals being excluded when assessing the MESI device compared to the Dopplex device.

### Device agreement

Ninety-nine systolic BP and diastolic BP MESI measurements (3 measurements for each of the 33 subjects), and 99 Dopplex systolic BP measurements, were available for further analysis. Table 2 compares mean BP values for the MESI and Dopplex with the corresponding Nissei reference value. The number of measurements differed from the Nissei reference by 5, 10 and 15 mmHg for systolic and diastolic BP, according to the ESH-IP, are summarised in Table 3 (MESI) and Table 4 (Dopplex). Based on these measurements, the MESI successfully passed part 1 of ESH-IP, Table 3. The Dopplex device passed the systolic BP requirements but failed part 1 due to a lack of diastolic BP readings, Table 4.

**Table 2:** Comparison of mean BP and numbers of participants classified as having low, medium or high BP based on comparison of the MESI and Dopplex systems with a calibrated reference device (Nissei). SBP = Systolic BP, DBP = Diastolic BP, SD = Standard Deviation.

	Nissei (reference)	MESI ABPI	Nissei (reference)	Dopplex ABIlity
**SBP (n = 33)**				
Mean	125.3	121.9	126.8	127.1
(SD, range), mmHg	(13.3, 104–150)	(15, 94–148)	(14.4, 98–164)	(16.2, 94–170)
Low SBP, n	18	22	21	18
Medium SBP, n	15	11	11	14
High SBP, n	0	0	1	1
**DBP (n = 33)**				
Mean	74.4	73.4	76.2	N/A
(SD, range), mmHg	(8.3, 54–90)	(8.7, 54–87)	(8.6, 62–92)	
Low SBP, n	22	22	22	
Medium SBP, n	11	11	11	
High SBP, n	0	0	0	

**Table 3:** Summary of the MESI validation according to the ESH-IP. The accuracy of the systolic BP (SBP) and diastolic BP (SBP) values is determined is based on whether values were within 5, 10 or 15 mmHg for 99 individual measurements (part 1) and then on a per-participant basis (part 2). The MESI device passed both part 1 and part 2 of the ESH-IP, suggesting this device is suitable for hypertension screening. The 2/3 and 0/3 represent the minimum number of comparisons within a 5 mmHg difference for systolic and diastolic BP, respectively.

PART 1 (99 measurements)	≤ 5mmHg		≤ 10mmHg		≤ 15mmHg		Grade 1	Mean (mmHg)	SD (mmHg)
Pass requirement	Two of	73	87		96				
	All of	65	81		93				
Achieved	SBP	64	89		99		Pass	4.7	3.5
	DBP	90	99		99		Pass	2.5	2.2
**PART 2(33 participants)**		**2/3 ≤5 mmHg**		**0/3 ≤5 mmHg**			**Grade 2**		**Grade 3**
Pass requirement		≥24		≤3					
Achieved	SBP	24		2			Pass		Pass
	DBP	31		0			Pass		Pass
**PART 3**									RESULT
									**Pass**

**Table 4:** Summary of Dopplex validation results according to the ESH-IP protocol. The accuracy of systolic and diastolic BP estimates is based on whether values were within 5, 10 or 15 mmHg for 99 individual measurements (part 1) and on a per-participant basis (part 2). The Dopplex device failed both part 1 and part 2 of the ESH-IP, suggesting this device is unsuitable for hypertension screening. The 2/3 and 0/3 represent the minimum number of comparisons within a 5 mmHg difference for systolic and diastolic BP, respectively.

PART 1 (99 measurements)	≤ 5mmHg		≤ 10mmHg		≤ 15mmHg		Grade 1	Mean (mmHg)	SD (mmHg)
Pass requirement	Two of	73	87		96				
	All of	65	81		93				
Achieved	SBP	66	93		99		Pass	4.3	3.2
	DBP	N/A	N/A		N/A		Fail	N/A	N/A
**PART 2 (33 participants)**		**2/3 ≤5 mmHg**		**0/3 ≤5 mmHg**			**Grade 2**		**Grade 3**
Pass requirement		≥24		≤3					
Achieved	SBP	21		1			Fail		Fail
	DBP	N/A		N/A			Fail		Fail
**PART 3**									RESULT
									**Fail**

The MESI passed part 2 of the ESH-IP requirements (Table 3). For the Dopplex, 21/33 participants had a minimum of 2 out of 3 measurements within 5 mmHg of the reference device. This was below the required target (Table 4). Therefore, the device failed this part of the ESH-IP protocol.

Part 3 of the ESH-IP combines the outcomes from parts 1 and 2 of the protocol. All of the requirements were satisfied for the MESI device (Table 3). The Dopplex device failed part 3 of the ESH-IP as it did not fulfil the requirements of either part 1 or part 2 (Table 4).

**Fig 1:**
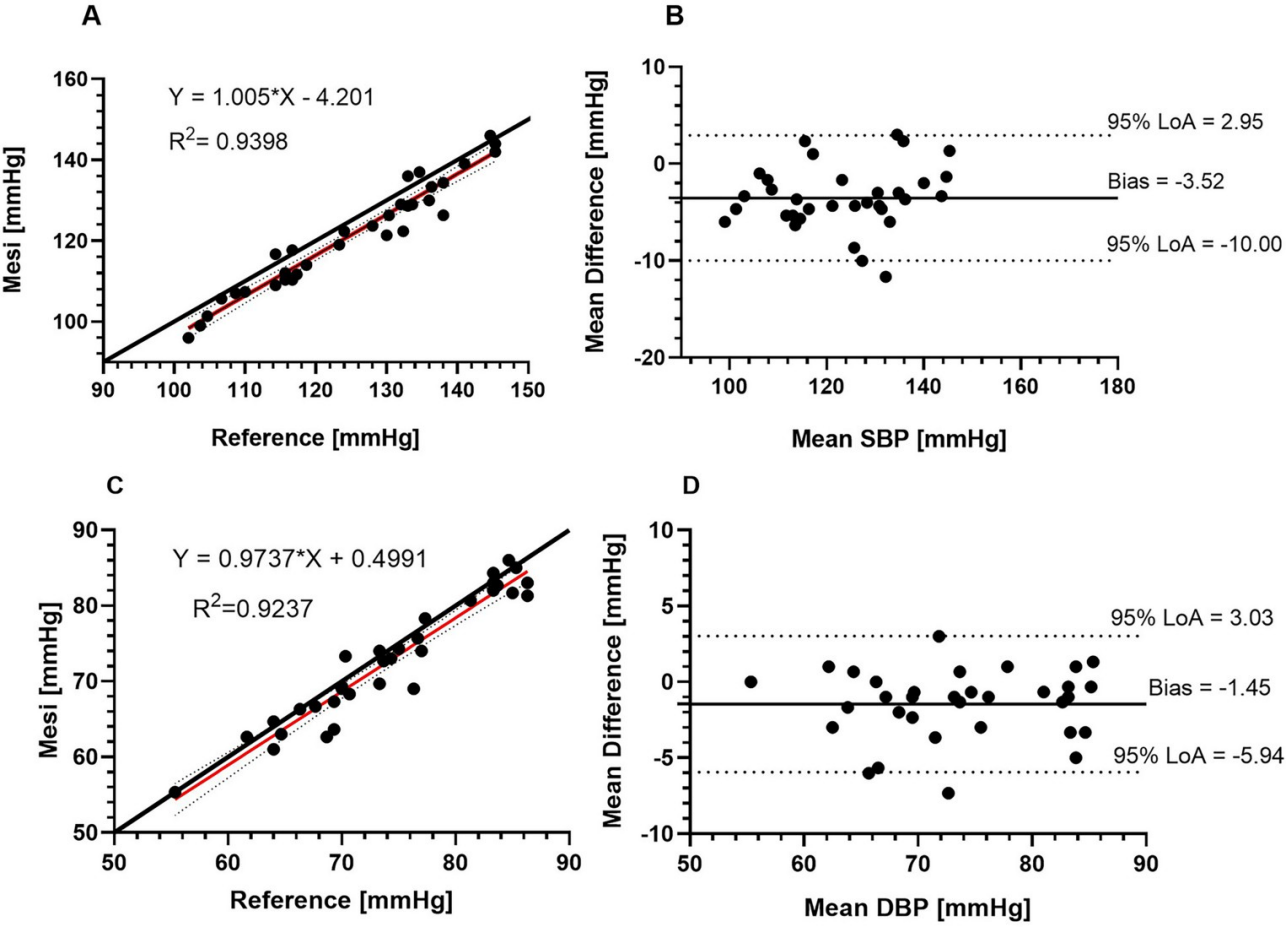
Agreement between the MESI and Nissei reference data, based on 33 pairs of systolic BP (A and B) and diastolic BP (C and D) measurements. The MESI tended to underestimate SBP by -3.5 (SD: 3.3) mmHg (95% LoA: -10.0, 3.0) and DBP by -1.5 (SD: 2.3) mmHg (95% LoA: −5.9, 3.0). The solid red line in A and C represents a linear fit to the measured data (circles), compared to the line of perfect agreement (black line).

**Fig 2:**
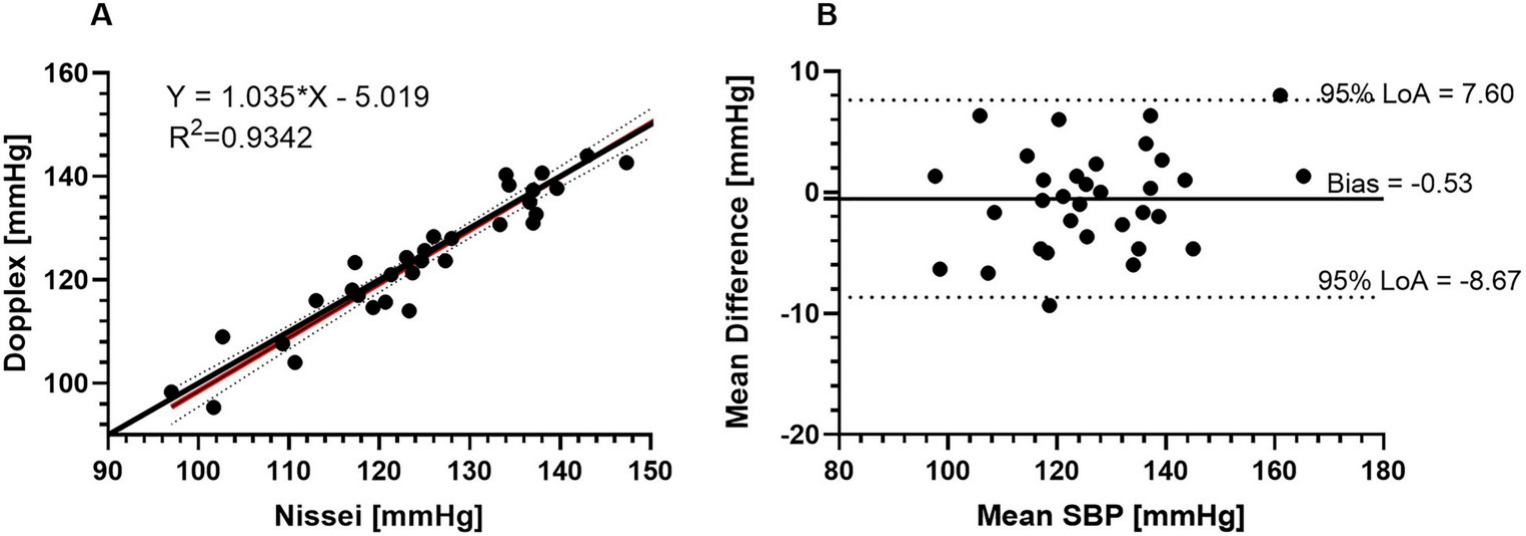
Agreement between the Dopplex and Nissei reference data, based on 33 pairs of SBP measurements. The Dopplex ABIlity was in close agreement with the Nissei reference device, with a bias of -0.53 (SD: 4.2) mmHg, but measurements were more variable with a wider 95% LoA ranging from −8.7 to 7.6 mmHg.

Linear correlation analysis assessed the association between test devices and reference measurements. As expected, systolic and diastolic BP estimates from the PAD and reference device were strongly correlated. Bland-Altman analysis revealed that the MESI underestimated BP with a mean bias (SD) of -3.5 (SD: 3.3) mmHg for systolic BP measurements and -1.5 (SD: 2.3) mmHg for diastolic BP, respectively (Fig 1). Bland-Altman analysis for the Dopplex device showed a mean bias of 0.5 (SD: 4.2) mmHg for systolic BP measurements (Fig 2).

## Discussion

This study is the first to validate the suitability of automated PAD devices for simultaneous PAD and hypertension screening. Both automated PAD devices trialled in this study have previously been reported to have high accuracy for PAD [14, 18]. Although PAD devices available on the market offer BP measurements, they are not licenced or validated for hypertension screening.

This study explored the suitability of PAD devices for hypertension screening by following the ESH-IP validation protocol. A mercury-free Nissei sphygmomanometer was used as a calibrated reference device which has a similar level of accuracy as that of a standard mercury sphygmomanometer.

Huntleigh’s Dopplex ABIlity did not meet the requirements of the ESH-IP as it failed to pass parts 1 and 2 for its SBP measurements and subsequently failed part 3. This device does not provide DBP measurements, preventing it from passing the accuracy criteria for DBP.

The MESI met the requirements of the ESH-IP. Bland-Altman analysis showed that the device slightly underestimates BP with a bias of -3.5 (SD: 3.3) mmHg (95% LoA: −10.0, 3.0) for systolic BP and bias of -1.5 ± 2.3 mmHg (95% LoA: −5.9, 3.0) for diastolic BP. The MESI BP data closely correlated with reference device estimates (SBP: R^2^ = 0.94 and DBP: R^2^ = 0.92, respectively). The MESI would be a suitable device for simultaneous PAD and hypertension screening in a national screening programme as an alternative to performing separate ABPI and hypertension screening tests.

## Limitations and future work

Our study had several limitations. Firstly, only one MESI device and one Dopplex ABIlity device were tested, which may not represent the performance of these devices overall. Two identical devices from the same manufacturer could potentially exhibit differences in readings. However, differences between devices would not impact our main finding that the Dopplex system would not pass the ESH-IP protocol. The next phase of this clinical trial involves assessing the suitability and accuracy of PAD and HBP screening when using multiple MESI devices in a population-based screening setting.

Since this was a healthy volunteer study, few participants exhibited raised BP. Most participants with clinically diagnosed hypertension were taking antihypertensive medication to control their BP. Further clinical trials using patients being screened for hypertension would be valuable for assessing the accuracy of this device in the very high BP range. Our findings are, therefore, only valid for medium to low BP values. Finally, the ban on mercury sphygmomanometers meant we had to use a mercury-free reference standard (the Nissei DM-3000). Using this device deviates from the ESH-IP validation protocol, however, previous researchers have suggested the Nissei to be a reliable, mercury-free alternative [12]. Two Nissei devices were used for this study. To confirm their accuracy, they were frequently compared and calibrated by the University Hospitals of Leicester NHS Trust Clinical Engineering Scientific Services team.

## Conclusion

The MESI ABPI MD device was sufficiently accurate for use in hypertension screening, according to the ESH-IP validation protocol. Huntleigh’s Dopplex ABIlity failed to pass the ESH-IP validation and is not currently suitable for hypertension screening.

## Supporting information

S1 Fig Two automated PAD detection devices: (A) MESI and (B) Dopplex. (TIF)

S1 Table Comparison between the two devices, MESI and Dopplex. (DOCX)

S2 Table Entry BP ranges for classifying low, medium and high blood pressure. (DOCX)

S3 Table Screening and recruitment details. (DOCX)
