# Supplementary figures and images for: Are portable ankle brachial pressure index measurement devices suitable for hypertension screening?
Source: PLoS One. 2023 Mar 21;18(3):e0283281. doi: 10.1371/journal.pone.0283281 (PMC10030014; doi:10.1371/journal.pone.0283281)

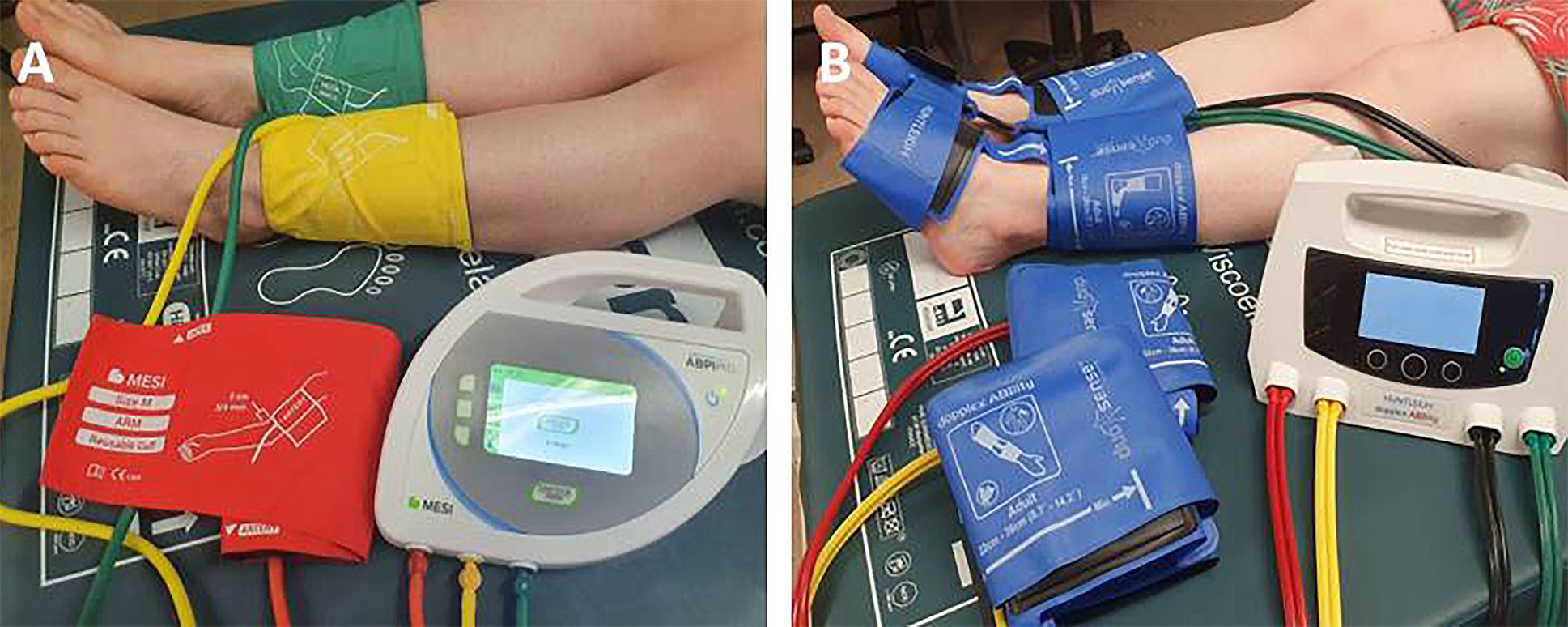

Supplement: S1 Fig — (TIF) [file pone.0283281.s001.tif]
