# Supplementary material for: Are portable ankle brachial pressure index measurement devices suitable for hypertension screening?
Source: PLoS One. 2023 Mar 21;18(3):e0283281. doi: 10.1371/journal.pone.0283281 (PMC10030014; doi:10.1371/journal.pone.0283281)
Supplement: S1 Table — (DOCX) [file pone.0283281.s002.docx]

**Supplementary**

**S1 Table .** Comparison between the two devices, MESI and Dopplex.

|  | MESI ABPI MD | Dopplex ABIlity |
| --- | --- | --- |
| Cuffs | Three colour-coded BP cuffs. Each cuff contains a diagram indicating an appropriate placement on each limb: the red cuff is for the upper arm, green for the right ankle, and yellow for the left ankle.  Cuffs are available in two sizes, medium and large. Simultaneous inflation of all three cuffs occurs to inflate the cuffs to the same pressure level. | Four BP cuffs, each comprised of two chambers (an upper and lower chamber). Each cuff is printed with an illustration demonstrating how the cuff should be placed. Cuffs are positioned around each arm and forearm, and around each ankle and foot. Wires connecting each cuff to the device is colour-coded: red (the right arm), yellow (the left arm), black (the right ankle), and green (the left ankle).  Cuffs are available in two sizes, medium and large, marked with the arm or ankle circumference. |
| Method of BP estimation | Oscillometric and volume plethysmography for recording brachial BP, for comparison with bilateral ankle pressure measurements. | Simultaneous volume plethysmography measurements. |
| Values reported | ABPI for the left and right sides of the body, SBP, DBP, and heart rate, on the device screen. | ABPI and pulse volume recordings for the left and right side of the body, and SBP, are displayed on the device screen. This device does not display DBP or HR. |
| ABPI estimation | Measurements recorded are the left ABPI, the right ABPI, and the upper arm BP, both SBP and DBP. | BP is measured in both arms before the higher pressure is used to calculate the ABPI. |
| Device validation | Clinical studies show that the MESI is effective at measuring ABPIs [15, 18] | Clinical studies show that the Dopplex is effective at measuring ABPIs [8, 14] |
| Device pressure range | The manufacturer reports the MESI to be capable of reading BPs between 0 and 299 mmHg (with a reported measurement accuracy of ± 3 mmHg, or 2%, whichever is smaller). The heart rate measurement range is 30 to 199 bpm with a reported measurement accuracy ± 5%. Measurement errors for the ABPI ratio estimate are reported to be within ± 0.1. | The manufacturer states that the device is suitable for measurements of brachial SBPs ranging from 80 to 220 mmHg and ankle pressures of 55 to 205 mmHg. The maximum cuff pressure is reported to be 230 mmHg. |
| Battery | Built-in lithium rechargeable battery. | Nickel-metal hydride battery. |
| Ease of use | Easy to use and portable. Identifies critical errors due to incorrect cuff placement or size, or patient movement during the measurement.  Connects to a PC to provide an electronic copy, or a printout, of the ABPI measurements with MESI results software. | Easy to use and portable. Identifies critical errors due to the cuff being incorrectly connected, cuff air leaks, inflation problems, excess pressure, internal faults, the SBP being out of a measurable range or the SBP cannot be calculated at all. ABPI and pulse volume recordings for the left and right side of the body can be printed from the built-in printer, along with SBP readings for each limb. There is a USB port for uploading readings to a computer.  Connects to a DR4 vascular reporting software package that provides electronic copy or a printout of ABPI measurements |
| Weight of device | Lightweight (weighs approximately 600 g). | Weighs approximately 3 kg. |
| Number of measurements between charges | MESI can take up to 50 measurements in between charges. | Dopplex provides up to 10 ABPI measurements in between charges. |
