# Supplementary material for: Are portable ankle brachial pressure index measurement devices suitable for hypertension screening?
Source: PLoS One. 2023 Mar 21;18(3):e0283281. doi: 10.1371/journal.pone.0283281 (PMC10030014; doi:10.1371/journal.pone.0283281)
Supplement: S2 Table — (DOCX) [file pone.0283281.s003.docx]

**Supplementary**

**S2 Table.** Entry BP ranges for classifying low, medium and high blood pressure.

|  | SBP^[[1]](#footnote-1)^ (mmHg) | DBP^[[2]](#footnote-2)^ (mmHg) |
| --- | --- | --- |
| Low | 90–129 | 40–79 |
| Medium | 130–160 | 80–100 |
| High | 161–180 | 101–130 |

1. SBP - systolic blood pressure [↑](#footnote-ref-1)
2. DBP - diastolic blood pressure [↑](#footnote-ref-2)
