# Supplementary material for: Are portable ankle brachial pressure index measurement devices suitable for hypertension screening?
Source: PLoS One. 2023 Mar 21;18(3):e0283281. doi: 10.1371/journal.pone.0283281 (PMC10030014; doi:10.1371/journal.pone.0283281)
Supplement: S3 Table — (DOCX) [file pone.0283281.s004.docx]

**Supplementary**

**S3 Table.** Screening and recruitment details.

|  | MESI ABPI MD | Dopplex ABIlity |
| --- | --- | --- |
| Total screened | 35 | 34 |
| Total excluded | 2 | 1 |
| Total on antihypertensive medication | 12 | 12 |
| Total included | 33 | 33 |
